# Segmental Plaque Heterogeneity in a Composite Right Internal Thoracic Artery–Radial Artery Graft as Visualized by NIRS-IVUS

**DOI:** 10.1016/j.jaccas.2026.108139

**Published:** 2026-05-05

**Authors:** Hiroki Saito, Kaito Yamada, Yuta Kagaya, Masanori Kanazawa, Masanobu Miura, Masateru Kondo, Hideaki Endo, Akihiro Nakamura

**Affiliations:** Department of Cardiology, Iwate Prefectural Central Hospital, Morioka, Japan

**Keywords:** atherosclerosis, coronary artery bypass, imaging, intravascular ultrasound

## Abstract

**Background:**

The internal thoracic artery has demonstrated well-established long-term patency in coronary artery bypass grafting, but the mechanisms underlying its durability remain incompletely understood.

**Case Summary:**

A man in his 70s presented with recurrent angina 23 years after bypass surgery, which had included a composite right internal thoracic artery–radial artery graft. One year postoperatively, a bare-metal stent had been implanted at the graft anastomosis. Near-infrared spectroscopy–intravascular ultrasound (NIRS-IVUS) showed minimal atherosclerotic change in the internal thoracic artery segment. The radial artery segment demonstrated superficial calcification without lipid accumulation, while lipid-rich neoatherosclerosis was confined to the stented anastomosis. The native right coronary artery contained extensive lipid-rich plaque and was treated with percutaneous coronary intervention. The patient was discharged without complications.

**Discussion:**

NIRS-IVUS revealed segmental differences in plaque within the composite graft.

**Take-Home Message:**

Composite arterial grafts may exhibit segmental plaque heterogeneity detectable by NIRS-IVUS.

## Visual Summary

**Figure:**
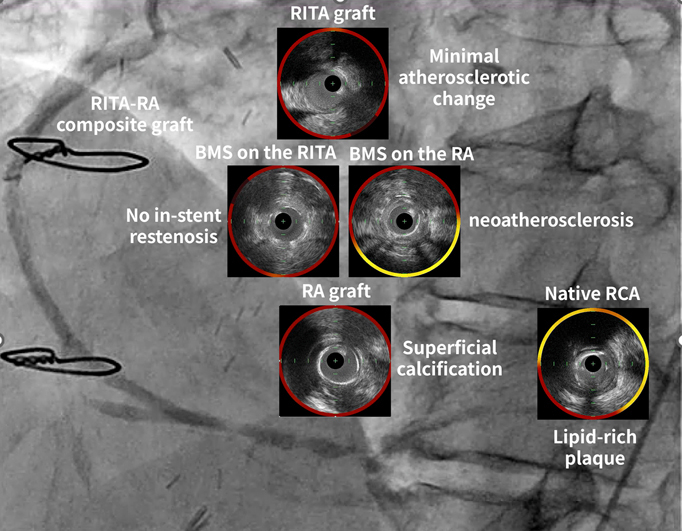

**Visual Summary:**
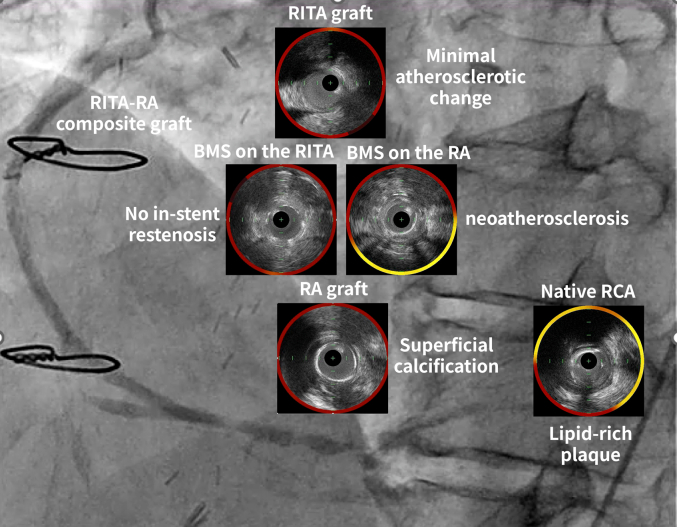
Schematic Illustrating a Composite RITA-RA Graft to the Native RCA With Representative NIRS-IVUS Images The right internal thoracic artery (RITA) segment exhibits no atherosclerotic changes or lipid-rich plaque. Within the bare-metal stent (BMS) placed across the RITA–radial artery (RA) anastomosis, the RITA side displays no evidence of in-stent restenosis or lipid accumulation, whereas the RA side demonstrates lipid-rich neoatherosclerosis. The RA segment also displays superficial calcification. The native right coronary artery (RCA) contains the culprit lesion, which is characterized by extensive lipid-rich plaque. NIRS-IVUS = near-infrared spectroscopy–intravascular ultrasound.

## History of Presentation

A man in his 70s with a history of coronary artery bypass grafting (CABG) presented with several months of worsening exertional angina. On admission, his vital signs were stable, and physical examination revealed no evidence of heart failure. The following day, he developed recurrent angina at rest. Emergency coronary angiography demonstrated progression of coronary artery disease, including a significant lesion in the left main–circumflex bifurcation, for which percutaneous coronary intervention (PCI) was performed. Although there was initial improvement, angina recurred the next day, necessitating PCI of the residual right coronary artery (RCA) lesion accessed through the right internal thoracic artery (RITA)–radial artery (RA) graft.

**Take-Home Message**

• Composite arterial grafts may exhibit segmental plaque heterogeneity detectable by near-infrared spectroscopy–intravascular ultrasound.

## Past Medical History

Twenty-three years prior, preoperative coronary angiography had revealed severe multivessel coronary artery disease. Findings included 99% stenosis with delayed antegrade flow in RCA segment #1, 90% stenosis in left anterior descending artery (LAD) segment #6, chronic total occlusion in segment #7, and 75% stenosis in the high lateral branch. Based on these findings, the patient underwent CABG for unstable angina with the following configuration: left internal thoracic artery (ITA) to the LAD, aorta to RA to the high lateral branch, and a composite RITA-RA graft to the RCA.

Because the in situ RITA was insufficient to reach the distal RCA, the RA was used as an extension to achieve complete arterial revascularization. This strategy was selected based on target vessel anatomy, conduit length and caliber, and intraoperative assessment of anastomotic geometry and anticipated flow characteristics. One year after CABG, significant stenosis at the RITA-RA anastomosis was treated with a bare-metal stent (BMS). The patient was evaluated routinely, without major cardiac events until the current presentation.

Comorbidities included diabetes mellitus, dyslipidemia, hypertension, and chronic kidney disease. The patient had a 36-year smoking history (1 pack per day) but had quit.

## Investigations

**Figure 1:**
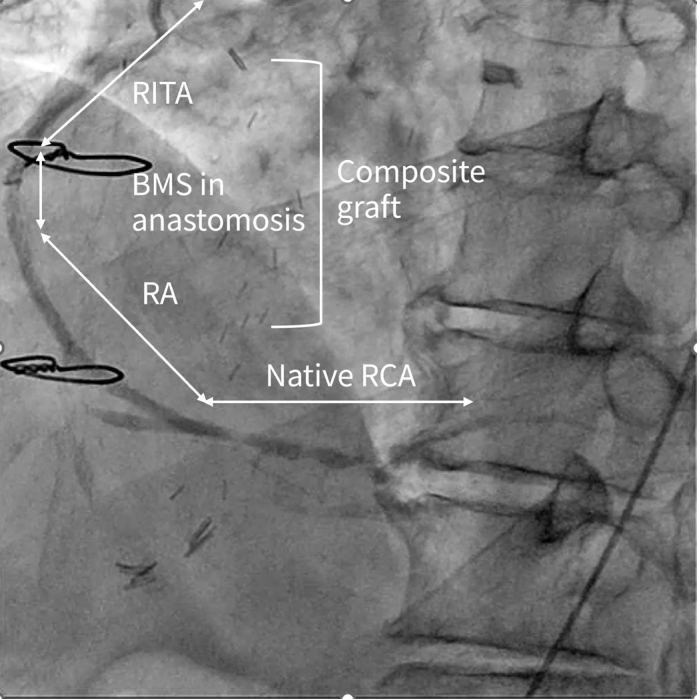
Coronary Angiography of the Composite RITA-RA Graft and Native RCA Coronary angiographic image demonstrating the composite right internal thoracic artery (RITA)–radial artery (RA) graft to the native right coronary artery (RCA). A bare-metal stent (BMS) is located at the anastomosis between the RITA and RA. The RITA, RA, and native RCA segments are labeled to illustrate the anatomical configuration of the graft conduit.

**Figure 2:**
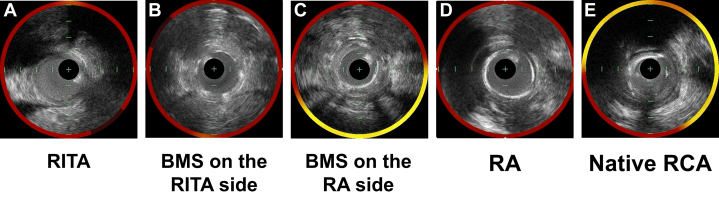
Representative NIRS-IVUS Images From the Native RCA and the Composite RITA-RA Graft Representative NIRS-IVUS images showing: (A) the RITA segment with minimal atherosclerotic change; (B) the BMS on the RITA side with no evidence of in-stent restenosis or lipid accumulation; (C) the BMS on the RA side demonstrating lipid-rich neoatherosclerosis; (D) the RA graft showing superficial calcification; and (E) the native RCA demonstrating extensive lipid-rich plaque.

**Figure 3:**
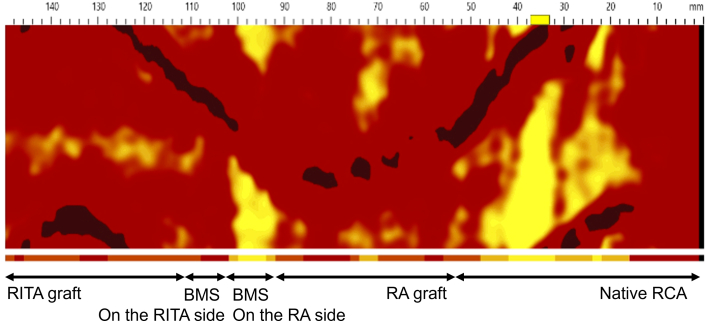
NIRS Chemogram From the Native RCA Through the Composite RITA-RA Graft A near-infrared spectroscopy (NIRS) chemogram was obtained by pullback from the distal native right coronary artery (RCA) through the composite right internal thoracic artery (RITA)–radial artery (RA) graft. Predominant lipid-rich plaque signals were observed in the native RCA, corresponding to the culprit lesion. A focal lipid accumulation was also noted in the RA segment at the site of the bare-metal stent (BMS) implanted at the RITA-RA anastomosis. In contrast, both the RITA and nonstented RA segments exhibited only minimal lipid signals.

During PCI for the native RCA lesion, near-infrared spectroscopy–intravascular ultrasound (NIRS-IVUS) imaging was conducted from the distal native RCA through the RITA-RA-RCA graft to assess the entire conduit (Figure 1). Selective RITA graft angiography to the native RCA is shown in Video 1. NIRS-IVUS demonstrated minimal atherosclerotic change in the RITA segment (Figure 2A). Within the BMS placed across the RITA-RA anastomosis, the RITA side exhibited no evidence of in-stent restenosis (ISR) or lipid accumulation (Figure 2B), whereas the RA side showed diffuse neointimal hyperplasia with lipid-rich plaque, detected as yellow signals on NIRS-IVUS (Figure 2C). The RA graft exhibited nearly circumferential superficial calcification (Figure 2D). The native RCA showed superficial calcification, regions of attenuated plaque, and lipid-rich plaque (Figure 2E). A video of the NIRS-IVUS pullback from the distal native RCA through the RITA-RA graft is available (Video 2). The NIRS chemogram obtained during pullback from the distal native RCA through the composite RITA-RA graft demonstrated predominant accumulation of lipid-rich plaque signals in the native RCA, corresponding to the culprit lesion. A focal lipid-rich region was also identified in the RA segment at the site of the BMS implanted at the RITA-RA anastomosis. No definitive evidence of lipid-rich plaque was observed in the nonstented RA segment. Likewise, the RITA segment demonstrated only minimal lipid-rich plaque along its entire length (Figure 3).

## Management

**Figure 4:**
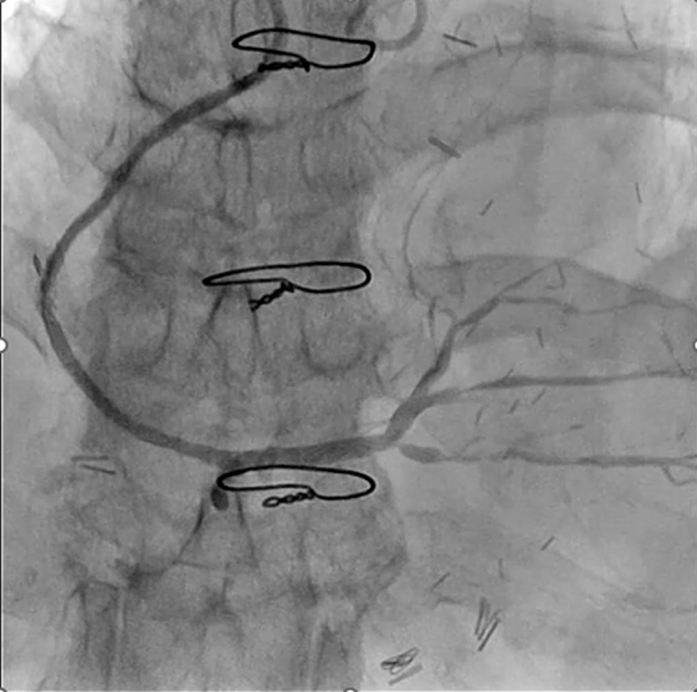
Final Coronary Angiography After Percutaneous Coronary Intervention of the Native RCA Final coronary angiography demonstrating successful revascularization of the native right coronary artery (RCA) via the right internal thoracic artery–radial artery graft. The culprit lesion in the native RCA was treated with stent implantation.

A drug-eluting stent was successfully implanted from segment #3 to #4AV (Figure 4). After the procedure, the patient remained asymptomatic and was discharged without complications.

## Discussion

The ITA is recognized for its superior long-term patency in CABG, particularly when used to bypass the LAD. When technical limitations are absent, the in situ left ITA is widely regarded as the conduit of choice for LAD revascularization.^1^ Its resistance to atherosclerotic changes contributes to the preservation of graft patency over time.^2^

Both the RA and ITA are classified as muscular arteries, characterized by relatively low elastic fiber content and abundant smooth muscle cells. This structure results in reduced compliance and potentially increased susceptibility to hemodynamic stress in high-pressure, high-flow environments such as the coronary circulation after CABG. However, the ITA possesses unique biological features. Notably, the it exhibits higher endothelial nitric oxide synthase expression and nitric oxide release than the RA, both at baseline and in response to stimulation, indicating a superior capacity for endothelial nitric oxide production.^3^ Increased nitric oxide bioavailability contributes to the ITA's resistance to atherogenesis by inhibiting lipoprotein oxidation, leukocyte adhesion, transmigration, and vascular smooth muscle cell proliferation and migration.^4^ These mechanisms support sustained endothelial function and long-term atheroprotection.

NIRS-IVUS reliably detects lipid-rich plaques and identifies vulnerable coronary lesions.^5^ In this case, NIRS-IVUS demonstrated that the RITA segment was free of atherosclerotic changes and lipid-rich plaque on both near-infrared spectroscopy and intravascular ultrasound. These findings indicate an absence of atherosclerotic progression in the RITA segment.

Local hemodynamics likely contributed to the asymmetric plaque distribution observed in this composite graft. Differences in vessel diameter, compliance, curvature, and downstream resistance between the RITA and RA can create segment specific shear stress patterns, resulting in more disturbed flow within the RA segment. Such flow environments are known to promote plaque development and may help explain the structural changes observed in the RA segment despite the graft's overall long-term patency.

ISR is primarily influenced by mechanical factors associated with the stent, such as metal-induced injury, local inflammatory responses, and changes in local hemodynamics, all of which contribute to neointimal hyperplasia. In contrast, native atherosclerosis is characterized by lipid accumulation, inflammation, and progressive plaque formation within an unstented vessel. The anastomotic stenosis observed in our patient 1 year after CABG likely resulted from technical factors at the anastomosis rather than intrinsic conduit biology. Therefore, the asymmetric ISR observed in this case cannot be attributed solely to biological differences between the conduits; local hemodynamic disturbances at the anastomosis must also be considered.

Although the ITA has demonstrated well-established long-term durability in multiple studies, the present case does not permit definitive conclusions regarding intrinsic biological superiority. Randomized data also support the long-term durability of the radial artery. In the RAPCO trial, the RA demonstrated higher 10-year patency than the free RITA,^6^ and 15-year clinical outcomes were similar or slightly favorable for the RA.^7^ These findings demonstrate that conduit performance is multifactorial and that superiority cannot be inferred from a single case.

This case illustrates a rare example of segmental differences in plaque characteristics within a composite RITA-RA graft visualized by NIRS-IVUS. The asymmetric distribution of neoatherosclerosis may reflect a combination of biological properties, technical factors at the anastomosis, and segment-specific flow environments.

## Funding Support and Author Disclosures

The authors have reported that they have no relationships relevant to the contents of this paper to disclose.

## Appendix

Video 1 Selective Angiography of the RITA-RA Graft Selective angiography of the right internal thoracic artery (RITA)–radial artery (RA) graft demonstrating flow to the native right coronary artery.

Video 2 NIRS-IVUS Pullback From the Native RCA Through the RITA-RA Graft Near-infrared spectroscopy–intravascular ultrasound (NIRS-IVUS) pullback performed from the distal native right coronary artery (RCA) through the right internal thoracic artery (RITA)–radial artery (RA) graft, demonstrating segmental differences in plaque composition.
